# Honeybee comb geometry emerges from local redistribution of angular space by worker construction activity

**DOI:** 10.1038/s44434-026-00004-2

**Published:** 2026-10-06

**Authors:** Vincent Gallo, Alice D. Bridges, Joseph L. Woodgate, Lars Chittka

**Affiliations:** 1 Department of Biological and Experimental Psychology, School of Biological and Behavioural Sciences, Queen Mary University of London, London, UK; 2 School of Biosciences, University of Sheffield, Sheffield, UK

**Keywords:** Neuroscience, Physics

## Abstract

Honeybee comb is unique among animal architectures in its strikingly regular hexagonal cells, yet how this geometry emerges remains debated. Do bees construct perfect hexagons from the outset, or does regularity arise through later adjustments? We examined comb-building dynamics by presenting honeybees (*Apis mellifera*) with wax stimuli that constrained cell placement and angular space. Across three experiments, we tested predictions about how bees distribute the available angular range at cell junctions, shape cell bases, and correct irregularities over time. Initially, cells were uneven, with curved walls and variable angles. When the angular space was restricted to 180°, bees built cells with 90° internal angles; as construction progressed and barriers eroded, angles shifted toward 120°, equalizing the 360° range among three adjoining cells. Similarly, opposed cells formed flat bases orthogonal to side walls (≈90°), contrasting with the pyramidal bases of natural comb. When separate tongues of comb merged, cells at junctions were highly irregular, but subsequent work significantly reduced disparities in wall angles and cell size. These findings reveal that honeycomb cell geometry emerges through a self-organizing process: bees iteratively redistribute space and adjust cell boundaries until equilibrium is achieved. This dynamic optimization explains the iconic hexagonal pattern without invoking complex cognitive strategies.

## Introduction

Honeybee comb has long been the subject of human fascination, with the first recorded analysis of its structure dating back to the ancient Greeks^1^. Honeycomb is characterised by both its regularity and its structural efficiency: a double-sided sheet of tessellated hexagonal cells that both minimises the wax required for construction and maximises storage capacity^2–4^. While the geometry of completed comb is well described, the process by which this geometry emerges from the construction activities of worker bees remains debated. We explore here whether comb regularity may be an emergent property arising from angular redistribution and equilibrium between the concurrent activities of builders in adjacent cells.

**Fig. 1:**
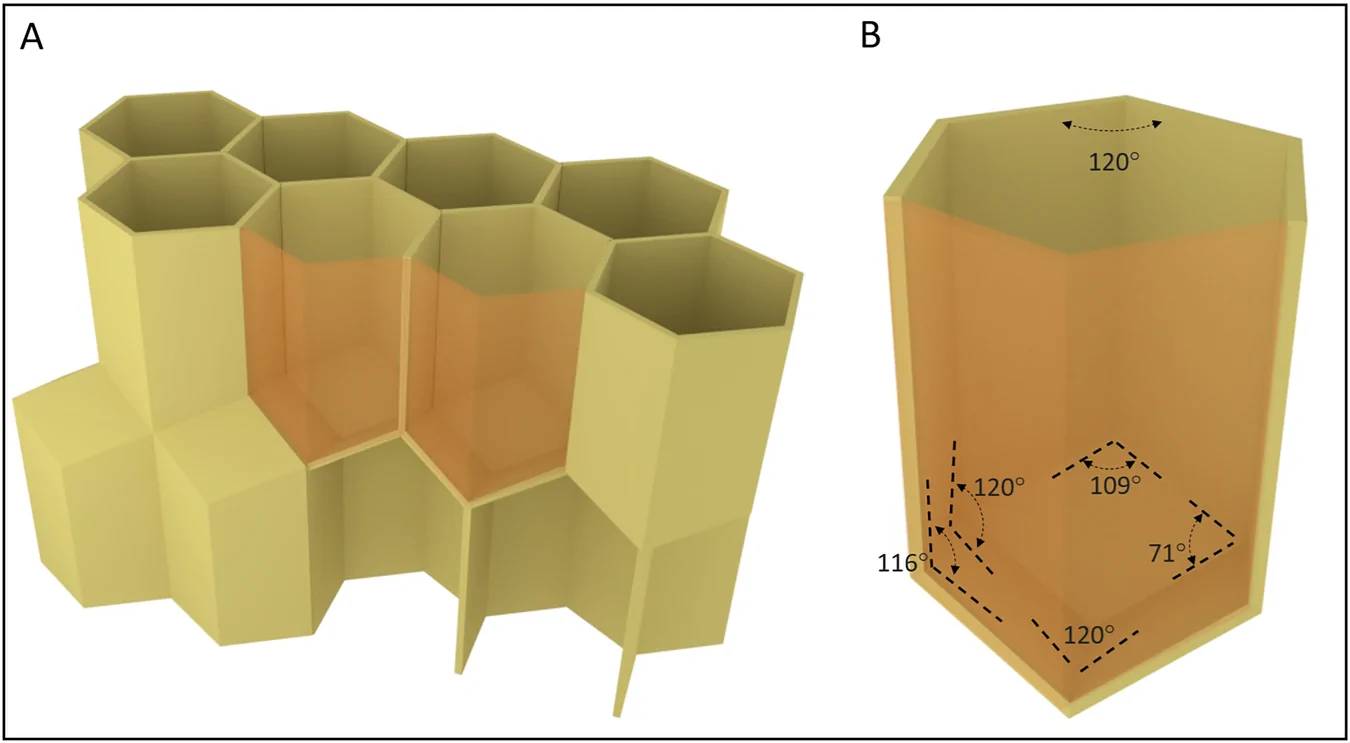
Geometry of honeybee comb. **A** Diagrammatic representation of cells within natural comb, where those on one face are offset by half a cell from those on the opposing face. Each base of a cell is formed of three rhombi, each of which in turn is part of one cell on the opposing side of the comb. Bisecting the rhombi at their lateral corners generates three triangles, which in turn form a pyramid with a (virtual) triangular base; hence the base of the comb cell is commonly said to be pyramidal. **B** Close examination of cells within natural comb reveals internal angles between faces of 120° in most cases. The dihedral angle (i.e. angle between two planes) between the long sides of a hexagonal comb cell and the side of rhombi at its base ranges from 116° to 120°. The internal angles of each rhombus are close to 109° (“major” or obtuse angle) and 71° (“minor” or acute angle) within the plane of the rhombus. The rhombi that form the base of the cell also meet at a 120 degree angle.

In natural honeycomb, the hexagonal cells have pyramidal bases that interlock along a shared backplane, resulting in an offset of half a cell between the two sides (Fig. 1A). Thus, while the hexagonal face of a sheet of comb is characterized by three-way cell intersections, with 120° wall junctions equally spaced in two dimensions around 360°, the interface between the two sides of comb involves *four*-way cell intersections involving a cell from one face centred at the intersection of three cells from the other. At each four-way intersection, the dihedral angles are also 120°, equally spaced around 360° in a three-dimensional layout (Fig. 1B).

Workers that add new cells undertake this work based on the structures of existing cells within the comb that they have already constructed^5–12^. Proper tessellation of cells along a single side of comb appears to require the placement of new cells between two extant cells, but as comb is double-sided, this actually requires new cells to be placed exactly between *three* extant cells. A necessary (albeit, insufficient) condition for the formation of regular hexagonal comb is, therefore, the correct positioning of each additional cell for which the bees are guided by the naturally occurring clefts between partially formed cells^13^. Such a guide is effective only where the earlier comb was already regular, and when the new cells are to be the same size as the existing ones. These conditions do not always apply, for example when changing from worker to the larger drone cells, attaching comb to a supporting structure, or when merging two sections of comb. Under these circumstances, the bees will be forced to construct a cell with uneven wall lengths, unequal internal angles and occasionally with fewer or more than six sides^14^. Indeed, descriptions and analysis of honeycomb that focus on its apparent perfection overlooks the fact that comb is only approximately regular^15^. Each cell, and indeed each wall of a cell, is formed subject to the builders’ ability to respond to local features, which invariably leads to local inaccuracies^16^.

Completed comb comprises cells with straight sides, yet during the early stages of construction, the cell walls are curved and their interiors are approximately circular, with walls straightening and cells becoming hexagonal only as construction continues^3,17,18^. We posit that honeybee construction workers, if building an isolated cell, would not construct a hexagonal prism with straight walls and a pyramidal base. Rather, they would create one as a circular tube with hemispherical base, similar to the curved, ovoid cells formed in the ground by species of solitary bees and within nest cavities by bumble bees^19,20^. However, honeybee cells are not isolated: they are densely packed, with each cell in direct contact with its neighbours. The ultimate hexagonal geometry of comb has previously been predicted to result from the compaction of cells that would otherwise have been curved^21–25^. We derived specific predictions to test this conjecture, and our own: that the even, regular shape of comb results from both the dense packing of cells and an equilibrium between the efforts of builders attending to each cell.

Some scholars have noted the similarity between the hexagonal honeycomb and some inorganic structures, such as graphene, or bubbles in bathtub foam (which sometimes form straight-lined boundaries), and have thus conjectured that honeybee comb naturally shapes itself into its familiar structure by nonbiological processes (e.g^17^). A precursor to this notion was already expressed by Kepler^21^. When considering a potential mechanism that could give rise to both the hexagonal outline of comb cells and their pyramidal bases (Fig. 1), Kepler^21^ used an analogy of peas being linearly constrained by their pod causing a flat face on each pea that runs orthogonal to the line of constraint. But honeybee comb does not “build itself” – its walls are constructed by individual worker bees attaching tiny slivers of beeswax to existing structures. Any newly constructed walls, or modifications of existing structures, come about by the activities by worker bees using legs and mandibles to extend and modify the construction. The question, then, is what rules these workers follow, and whether, for example, they possess innate behaviour routines that result in building small planes with 120^o^ angles between them.

We propose that construction workers, as a rule, endeavour to form a cylindrical or ovoid cell, with the perimeter of the cell established at an ideal radius. However, where such attempts interact with those of other builders working on adjacent cells, the initial conjunction will be uncoordinated and thus asymmetric. Only after further work will a balanced distribution of space between the conjoined cells emerge. Cell walls within completed comb typically have a cell on either side, and therefore will have been subject to the same influence exerted from both cells. Equilibrium in this case would be a flat wall and junctions where these walls meet typically arise through contact between three, and occasionally four, cells (Fig. 1^15,16^;). The construction of a cell both influences and is influenced by the construction of an adjacent cell, and therefore any walls meeting at a junction will eventually be arranged such that the available angular range (360°) is divided equally between all cells sharing the junction. In the case of three cells meeting at a junction, the internal angle of each would eventually settle at ~120°, as is widely observed in natural honeycomb.

We thus posit a generative principle: individual workers tend to extend or excavate wax to form quasi cylindrical cells, but where neighbouring workers act on adjacent cells, mutual constraints lead to iterative redistribution of both angular space and material. This redistribution proceeds until local equilibrium is approached, such that the available angular range at a junction is approximately equally apportioned among the cells that share it. Under this framework, outcomes (angles, base forms) are consequences of local constraints, not the result of innate behavioural rules such as “build 120° angles everywhere”. We test three separate predictions in this study.

Prediction 1 – the internal angle between two walls of a single cell is not *necessarily* 120°. Rather, we predict that the rule used by construction workers is to subdivide the available angular range around a junction equally between the cells. e.g., if space is constrained to just 180°, workers will erect a new wall so that two cells meeting at a junction will each have an internal angle of 90°.

Prediction 2 – in a similar vein, the angle between the base of a cell and its side walls is not necessarily 120° as in the normally pyramidal bases. Rather, where experimental manipulation causes cells to lie directly opposite each other on the two sides of the comb, workers' construction activities will retain the mutual base as a single face at a 90° angle to the side wall for each cell.

Prediction 3 – irregular cells (such as occur when two neighbouring comb constructions are merged) will show reduced variance in internal angles and convergence of cell areas after further construction, with the magnitude of change scaling with initial irregularity, in line with building rules derived from predictions 1 and 2.

To test these predictions, we offered honeybees atypical workpiece conditions in the form of specially designed wax stimuli, which they were allowed to build upon. The resulting construction was observed at an early stage, and again once further work had been undertaken.

## Results

*The available angular range around a junction was evenly divided between the cells meeting at that junction*.

**Fig. 2:**
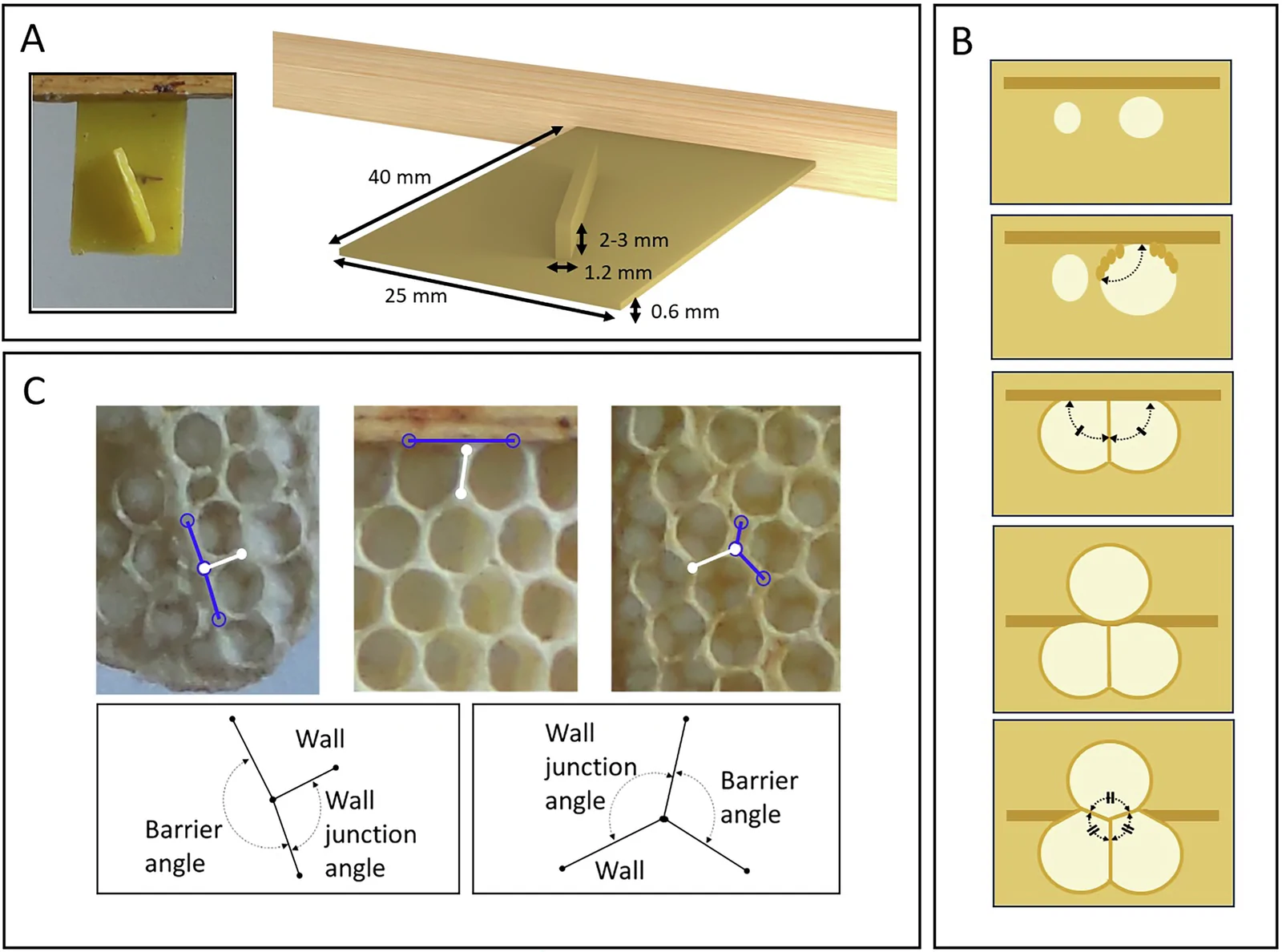
Design for Experiment 1. **A** Linear barriers were welded perpendicular to the wax tab. **B** Predicted progression of cells built against a barrier. Top: Initial construction close to the barrier will grow until a cell is built, exploiting the barrier as one of its walls. Second from top: An early-stage cell with peripheral wax deposits that form the basis of subsequent walls. Centre: As construction continues, a second cell is built adjacent to the first against the linear constraint, with a mutual wall arranged to balance the two equal internal angles at 90°. Second from bottom: Further construction on the opposite side of the barrier will result in its erosion. Bottom: The influence of this third cell results in the available angular range (now a full circle) being divided evenly between the three cells. **C** Example measurements taken of wall-to-wall angles. Top Left: A wall between a pair of cells built against a wax barrier. Top centre: The wall between a pair of cells built against a fixed barrier: the wooden frame. Top Right: A later image of the same cells shown in Top Left, taken after further construction during which the workers re-aligned the cell walls. Bottom Left: The measured attributes of the feature shown in Top Left: the barrier angle and the wall to barrier angle. Initially the barrier angle would have been 180°, but in the case of the wax barrier, some change resulted from the construction of the abutting cells. Bottom Right: Measurements taken after further construction, as shown in Top Right, where the barrier had been eroded.

In Experiment 1, we measured the corner junctions at a wall under three conditions as follows: “Paired” where the wall is between two adjacent cells that were constrained by a wax barrier (Fig. 2A, B); “Frame” where the wall is between two adjacent cells from the top row of comb that were constrained by the wooden frame, and “Triple” where cells were initially built against a wax barrier that had been eroded as construction progressed (Fig. 2B, C).

The stimulus comprising a wax barrier restricted the available angular range to 180° at the junction between two cells built against the barrier. As construction progressed, including cells built on the other side of the barrier, the wax barrier was eroded, and junctions between three cells would emerge with an available angular range of 360°. If our prediction was correct, two internal cell angles at the junction (one either side of the wall between the cell pair) would be recorded as ~90° when two cells were constrained by a rigid barrier but slightly more for a wax barrier, depending on the degree of erosion at the time of measurement. Where the barrier had been substantially eroded, and had been fashioned into two walls of a third conjoined cell, the angular distribution would be closer to 120° for each of the three cells to meet at a junction.

**Fig. 3:**
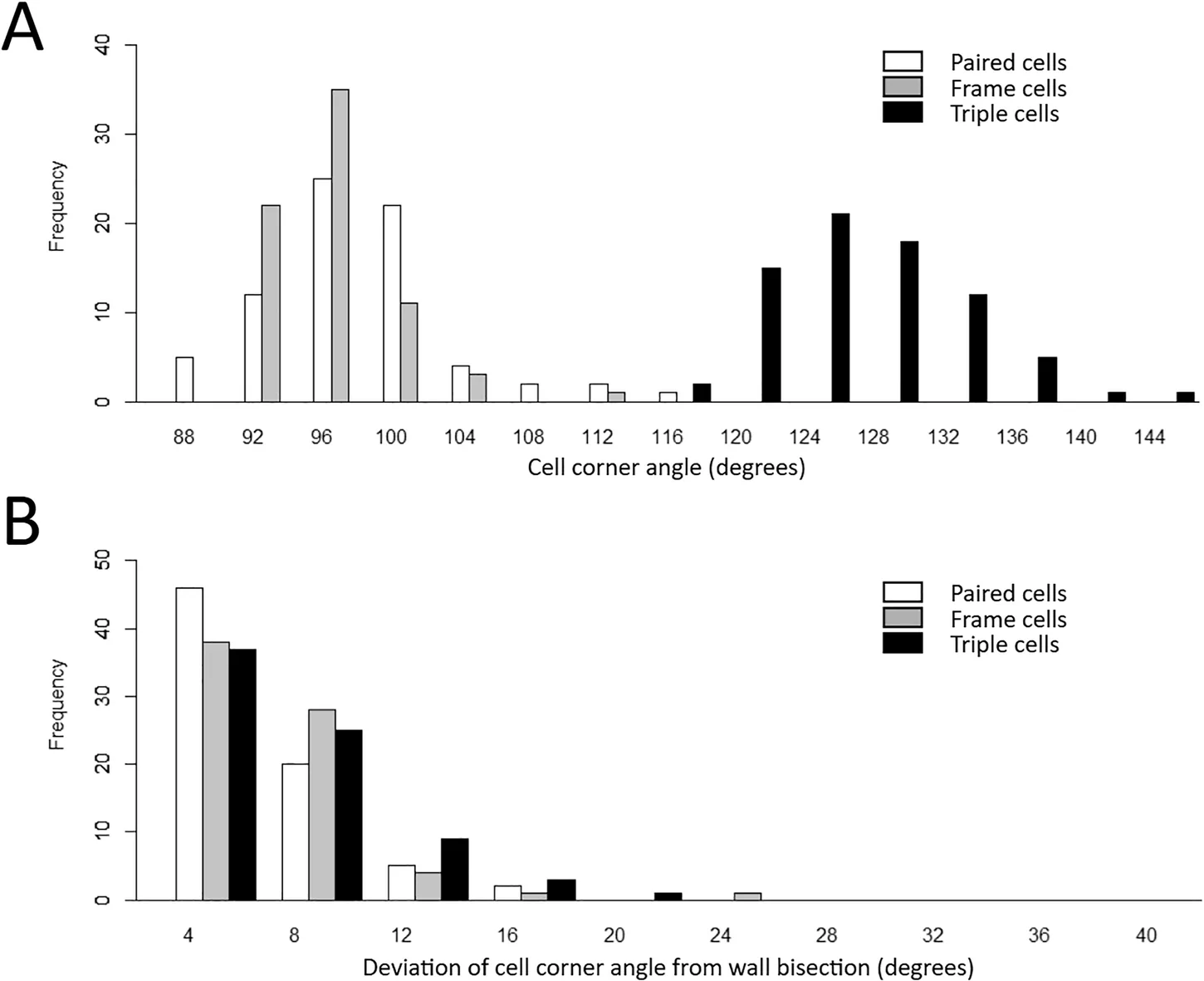
Cell corner angle measurements. **A** Histograms, with a bin size of 4 degrees, showing the distribution of the larger angle at cell corners in three configurations. Adjacent cells against a linear constraint, i.e. ‘paired cells’, are pairs of cells that share a wall abutting a wax barrier where the angle was measured between the mutual cell wall and the constraint (*N* = 73, 95.34° ± 5.37°. Data are presented as the mean ± standard deviation throughout). Adjacent cells built against the frame, offering a wooden linear constraint, are labelled ‘Frame cells’ where the measurement was the larger angle between the mutual cell wall and the wooden frame (*N* = 72,94.13° ± 3.56°). The wax constraint, that initially gave rise to the ‘paired cells’, was eroded following further work by the bees leading to the construction of a third cell, typically at the junction where the initial mutual cell wall joined the barrier resulting in a junction of three cells walls, labelled ‘Triple cell’, for which the angle between each cell’s walls at such a 3-way junctions was measured (*N* = 75, 124.49° ± 5.49°). **B** The deviations of those walls from the predicted, balanced, placement of the cell walls were 3.84° ± 3.11°, 4.21° ± 3.54° and 5.00° ± 3.92°, “paired”, “frame” and “triple”, respectively.

The larger of the two corner angles at a wall dividing “paired” cells (*n* = 73) were measured as 95.34° ± 5.37° and the measurements taken from the “frame” cells were similar (*n* = 72, 94.13° ± 3.56°; Fig. 3A). The measurements from “triple” cells, where the initial wax barrier had eroded and resulted in three cells sharing a 360° angular range, were markedly different from those for the “paired” (*n* = 75, 124.49° ± 5.49°, *T*_146_ = 32.65 *p* < 0.00001; Fig. 3A).

A Kruskal-Wallis test was conducted to determine whether the displacement angles measured between a cell wall and the line of bisection, derived from the barrier associated with each cell arrangement, differed between the three categories of cell arrangements: “paired”, “frame” or “triple” (Fig. 2). No significant differences were found among the three categories (Fig. 3B; Chi square = 3.43, *p* = 0.18, df = 2). Taken together, these results demonstrate that the angles between cell walls equalised the distribution of the available angular range between cells meeting at a junction. Furthermore, when the angular range changed as construction progressed, the sizes of cell corner angles were adjusted to maintain this equilibrium, as predicted.

*Single-faceted bases were built orthogonally to the walls of two opposing cells*.

**Fig. 4:**
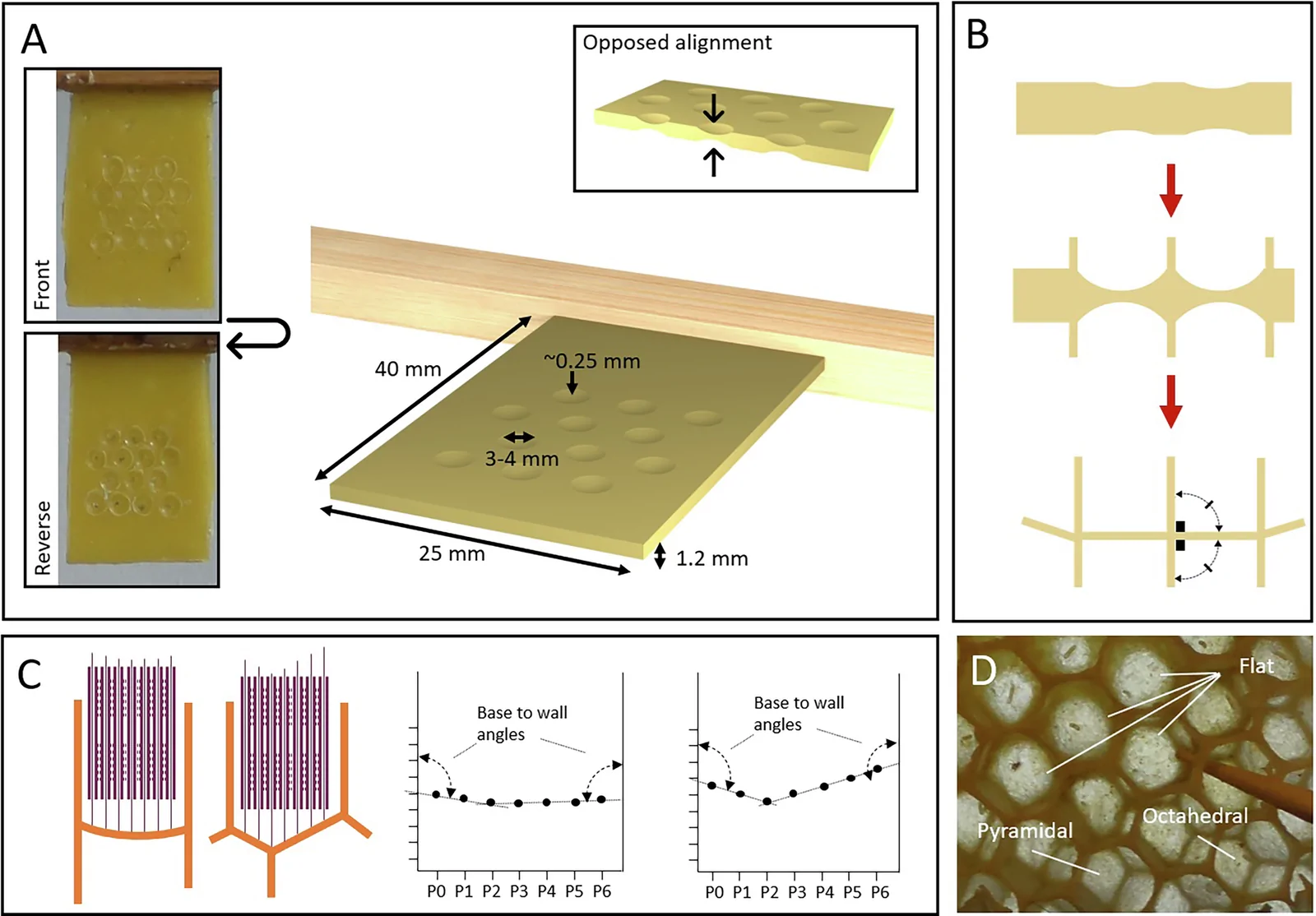
Design for Experiment 2. **A** Pits were pressed into both sides of the wax tab used for Experiment 2. The pits were arranged so that they were directly opposite each other (see inset). **B** Predicted construction of flat-bottomed cells when these are aligned in opposition on either side of the comb. Top: Cross section through the wax tab carrying the initial indentations, which guide the alignment of cells. Centre: Expected progression of construction, showing initial cell formation via the excavation of the guide pits and deposition of wax at their edges. Bottom: As construction continues, the substrate is thinned and the cell walls extended. Cell location, guided by the initial indentations in the stimulus, would lead to cells aligned on opposite faces of the comb and, for those cell pairs, the 180° between opposing cell walls would be divided equally between the cells. This would result in the mutual base being placed orthogonal to the cell walls. **C** Measurement of the base profile for aligned cells and for naturally displaced cells using a compound depth gauge comprising seven probes Left: The relative depth at each of the seven points was then used to compute a pair of regression lines and thus the base to wall angle at each side of the base, illustrated for a ‘flat’ base (Centre) and for a pyramidal base (Right). **D** Selection of flat-bottomed cells and some unusually shaped ones (in transmitted illumination). Of these, the cells marked “flat” were selected for measurements, but others in the image were rejected as they showed insufficient overlap (<80%).

**Fig. 5:**
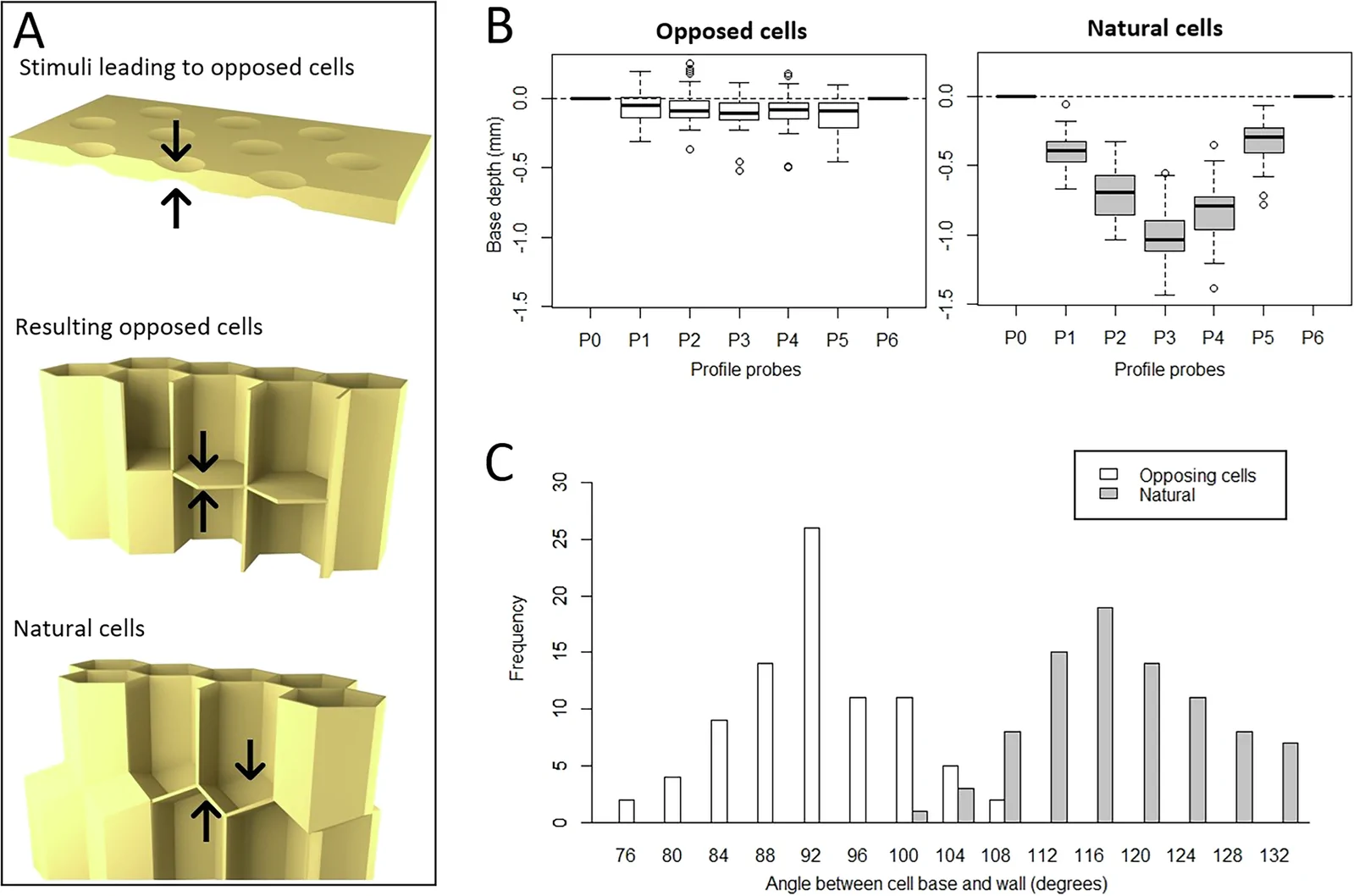
Comparison of opposing and natural (non-opposing) cell bases. **A** Profiles of opposed and natural cell bases. An artificial base with concave structures aligned on both sides (top) led bees to build opposed cells where walls of the hexagonal columns also aligned (middle). In this scenario, the base of the cells was at right angles to the cell-walls, while natural cells had pyramidal bases with a ‘V’ shaped profile (bottom). **B** Measuring the profile of the cell base with a purpose-built probe allowed us to quantify this difference: bases of opposed cells tended to have minimal depth of under 0.2 mm, whereas natural cells had a depth of close to 1 mm at the centre. **C** The distribution of angles between the base of a cell and its side wall, shown for opposed and natural cells. The samples of opposed cell bases (*N* = 84, 90.3° ± 6.7°) had a distinctly different distribution compared with those for natural cells (*N* = 86, 116.2° ± 7.5°).

In Experiment 2, we presented bees with wax tabs with indentations pressed into both sides, arranged to be opposite each other (“opposed cells”; Fig. 4A, B). This differed from natural comb, where cells on each face are offset by approximately half a cell. We took measurements from 42 opposed cell bases and 43 natural cell bases using a multi-pronged depth probe (Fig. 4C) and found a marked difference in their shape (Fig. 5). The box plots show the height for each probe point relative to the ends, with median, interquartile range and outliers depicted.

For the opposed cells, the maximum distance of a point from a line of regression through the 7 points on the profile was 0.13 mm ± 0.06 mm, which was significantly less than that measured for natural cells (0.58 mm ± 0.11; *t*_(65)_ = 22.59, *P* < 0.00001; Fig. 5A, B). Our prediction was that the angle between the base of a cell and its walls is not necessarily 120°, but is instead distributed equally between adjacent cells where the base forms the common wall: if this prediction were correct, then we would expect to see 90° angles in opposed cells, and 120° angles in “natural cells”. The average slope of a base relative to the side walls of its cell for opposed cells was 90.3° ± 6.7°, which differed significantly from the values found for each side of a base of a natural cell (116.2° ± 7.5°, *t*_(65)_ = 22.59, *P* < 0.00001; Fig. 5C). To determine whether the mean angle was equivalent to the predicted value of 90°, we performed a *t*-test with the null hypothesis that there is no difference between the sample and the predicted value (*t*_(83)_ = 0.42, *P* = 0.673, the null hypothesis should not be rejected) and two one-sided *t*-tests, with null hypotheses of the prediction plus SESOI and prediction minus SESOI, respectively. We used a SESOI of 0.5 Cohen’s *d*, assuming a moderate effect. The null hypotheses for both are rejected (the largest *p* value was *t*_(83)_ = -4.16; *P* = 0.000039; the interval of equivalence was 0.23). Hence the angle between the base and the side wall for opposed cells was found to be equivalent to the predicted value of 90°.

*Irregular cells formed at the intersection of discrete sections of comb were altered as construction progressed, becoming more regular*.

**Fig. 6:**
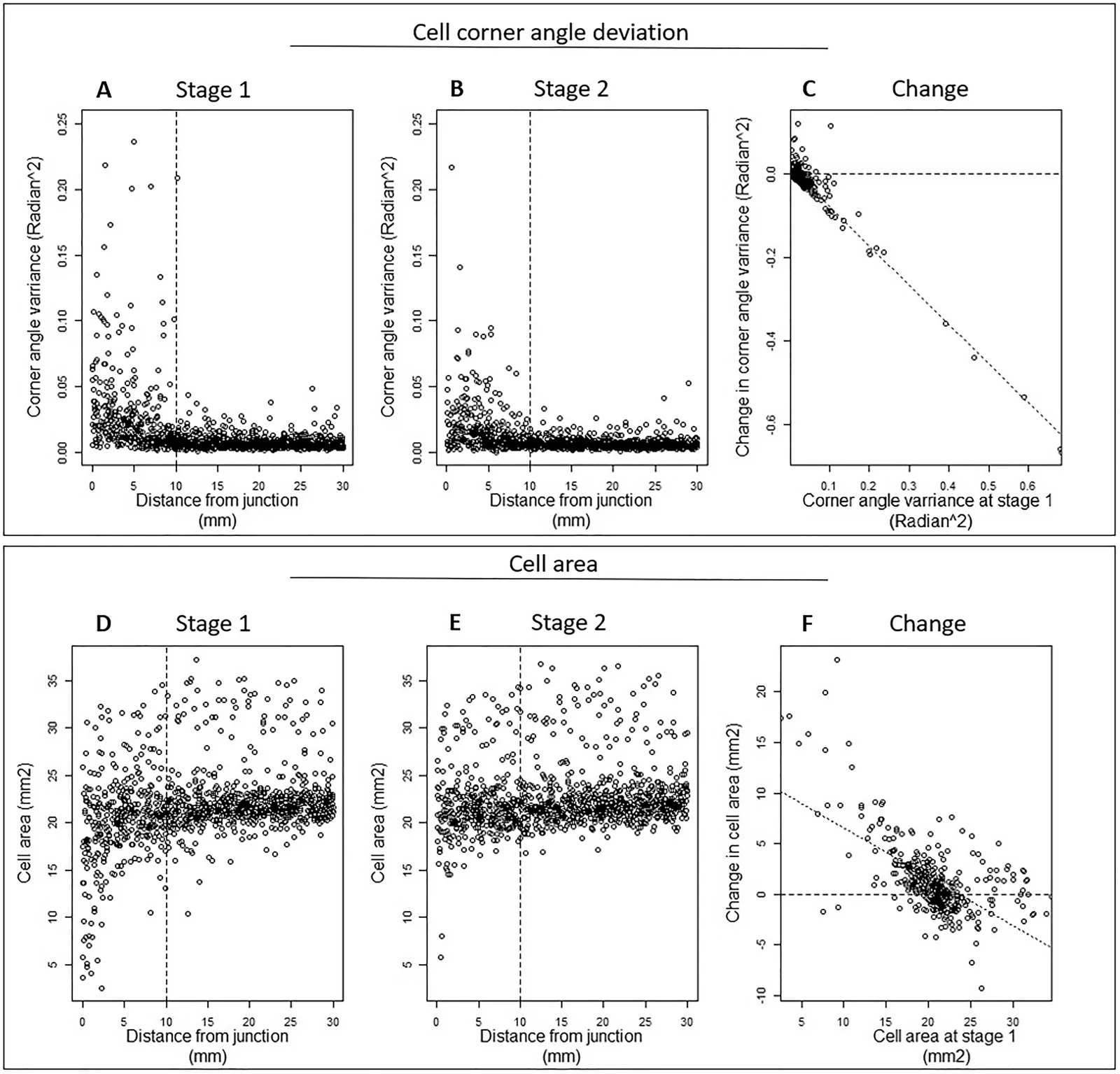
Distribution of cell features, compared at stage 1 and 2 of construction. Scatter plots for measure of cell regularity at stage 1, when tongues had just touched; at stage 2, after further construction and the change (value at stage 2 minus that at stage 1) for each cell. **A**, **B** Variance of intra-cell corner angles as a function of the orthogonal distance from the line where the two tongues met. **C** The change in cell corner angle variance from stage 1 to stage 2 as a function of corner angle variance at stage 1. The change in irregularity was significantly correlated with the measure at stage 1. The points are overlaid by a regression line relating the values and indicating that the more irregular cells underwent greater improvement. **D**, **E** Cell area, as a function of distance from the tongue junction. Cells close to the line of contact show a greater degree of irregularity; more variability in the internal corner angle and a wider range of cell sizes with more undersized cells. Points to the left of the dotted line are from cells within 10 mm of the junction for which the changes were computed. **F** the change in cell area (area at stage 2 – area at stage 1) as a function of the cell area at stage 1. The change in cell area was significantly correlated (see main text) with the cell area measured at stage 1. The points are overlaid by a regression line (dotted) relating the values, indicating that undersized cells were enlarged while oversized ones were made smaller.

Experiment 3 was designed to test whether initial uneven distributions of available space would be redistributed more evenly between adjacent cells as construction progressed. Here, we focused on the merging of distinct tongues of comb, as cells around the line where tongues meet are likely to exhibit a high degree of irregularity—unequal internal corner angles within a cell and uneven areas of cells within the sample population. We took measurements from cells within 30 mm of the line of contact where two tongues touched, with a total of 1068 cells measured at stage 1 (shortly after contact with the neighbouring comb) and 1048 cells measured at stage 2 (following further construction by the bees; note that the reduction in cell number was due to the fact that the reworked, more regular comb, had cell centres that shifted; and where they had moved, fewer cells fell within measurement boundaries). Figure 6 shows how the geometric characteristics of cells (intra-cell corner angle variance, Fig. 6A–C, and cell area, Fig. 6D–F) indicate a greater degree of irregularity for cells closer to the junction, where comb tongues came into contact. At Stage 1 there were 373 cells close to the line of contact (within 10 mm) and 357 at stage 2 (again, fewer fell within the selection rectangle). The location of each cell at each stage was used to match cells between images and so determine the change from stage 1 to stage 2 for individual cells. Cells selected for analysis were those close (<10 mm) to the junction. This value was chosen to include cells that would have been partially constructed at the edge of each tongue, prior to them coming into contact. For cells close to the junction (<10 mm) we were able to pair 350 of the 373 cells (93.8%) at stage 1 and of the 357 cells (98.0%) at stage 2.

The 350 paired cells within 10 mm of the junction had mean variance of internal angles at stage 1 of 0.0355 rad^2^ ± 0.072 and by stage 2, this had become 0.0182 rad^2^ ± 0.0209.

Simple linear regression was used to test if initial cell angular irregularity significantly predicted the change in angular irregularity between stage 1 and stage 2. The overall regression was statistically significant (*R*^2^ = 0.9167, *T*_350_ of 13.18 and *P* < 0.0001). Angular regularity was improved in proportion to the initial degree of irregularity (*β* = −0.941, Fig. 6C).

The 350 paired cells had mean area at stage 1 of 20.52 mm^2^ ± 4.7. By stage 2 this had become 21.98 mm^2^ ± 3.77, where 226 cells (65%) had increased in size. Simple linear regression was used to test if the initial cell area significantly predicted the change in cell area between stage 1 and stage 2. The overall regression was statistically significant (R^2^ = 0.389, T_350_ of 16.68 and *P* < 0.0001). Undersized cells at stage 1 were enlarged by stage 2 and oversized cells at stage 1 were made smaller by stage 2 (*β* = −0.482, Fig. 6F).

## Discussion

Honeycomb, when completed, has a well-characterised and appealing form comprising regular hexagonal cells that lie in clear, nearly straight rows and combine to form a structure that has the appearance of being both strictly-defined and intentional. This appearance has led many scholars to ask how the bees form such a regular structure^21,23,24,26,27^. Kepler^21^ stated that a hexagon, giving honeycomb cells their internal angles of 120°, is simply the polygon best suited to the tessellation of cells across the plane of honeycomb. Kepler also pointed out that the three rhombi at the base of each cell might be imagined as being three of the twelve rhombic faces of a rhombic dodecahedron, a regular solid with internal angles of 120°.

The classical accounts by Kepler^21^, Huber^27^ and Darwin^23^ emphasised the efficiency of the tiling properties of hexagons and rhombic shapes at the base of comb cells of natural honeycomb. But how do such structures arise from the construction activities of individual worker bees? Our data suggest that neither specific angles or forms need to be explicitly targeted; rather they emerge when adjacent builders iteratively equalise the available angular range at junctions. In our experiment 1, and indeed where the angular range was restricted to 180°, new wax walls were constructed perpendicular to existing walls, resulting in corner angles of 90°.

The base of a cell, comprising three rhombic faces that are each shared with a cell on the opposite face of a blade of comb, is shaped as a triangular-based pyramid (Fig. 1). This form could be of any depth with suitable angles within each rhombus to maintain the pyramidal geometry of the cell base. For example, the internal angle of the rhombi at the meeting point in the centre of the cell, could be large, with angles of almost 120° in the case of a nearly flat base. However, a base of increased depth would contain rhombi with smaller corner angles, with those of an extremely deep, pointed, base approaching 0°. The major (obtuse) angle of a base rhombus within natural comb was measured to be 109° 28’^27,28^. Réaumur^29^ calculated that an angle of 109° 26’ would maximise storage volume while minimising the quantity of wax required for construction. Rather than seek a purposeful cause for the geometry, our research shows that the observed geometry arises naturally for triplets of cells positioned within an angular range of 360°, and an even distribution therefore causes each cell to occupy 120° of this range. When applied to three cells in a 2-dimensional arrangement, the result is a triple junction within the tessellated hexagons, as is visible on the face of the comb. However, when the junctions are similarly equalised at a cell base between four cells in a 3-dimensional arrangement, our results show that the bees again equalise the angular distribution, giving rise to internal angles of 120°. The cell bases will therefore adopt the shape of three adjacent faces of a regular rhombic dodecahedron, within which each dihedral angle (angle between adjacent planes) is 120°, and the shape of the major (obtuse) angle for each rhombus is 2 * Cos^-1^(1/√3) or 109.47122° (109° 28’ 16”). This implies that honeybees do not construct comb in a manner aimed at maximising efficiency; rather, the more parsimonious explanation is that they simply balance the distribution of angular space between adjacent cells.

Most natural combs built by honeybees will contain cell bases that are exactly, or close to, a section of a rhombic dodecahedron. However, the results of experiment 2 demonstrate that bees can also build abnormal cells with flat bases. Our prediction was that the cell internal corner angle would be an integer fraction of the available range, not that every angle within a cell will be 120°. Thus, for a range of 180°, split between two cells, the wall angle should be 90° - this was the outcome of experiment 2. Flat bases were formed where cells lay in direct opposition, while normal rhombic bases were formed where cells overlapped by half their width, with the centre of a cell on one face positioned opposite the triple junction on the other face. Our prediction addresses both situations. For alternative arrangements of cells, the same mechanism will yield different base structures; hence, the geometry and arrangement of base faces is a consequence of the alignment of cells on either side of the comb. Figure 4 shows a range of base forms, including flat bases (Fig. 4D), pyramidal bases (Fig. 4D) and some that approximate a subsection of a truncated octahedron, a solid shape comprised of 14 faces (6 squares and 6 hexagons; Fig. 4D). Tóth^30^ suggested that such a shape might offer improved efficiency. In all cases, each facet of the base would be formed to cover the area where a cell on one face overlaps a cell on the opposite face of the comb.

Alteration of cells subsequent to their initial formation has been observed previously, albeit not explained. For example, this was seen when bees built comb upon wax foundations where the indentations were of a scale inappropriate for the species of honeybee^31^. The authors found that *A. mellifera* built cells on foundations with indentations that were spaced too closely (being appropriate instead for *A. cerana*, a smaller species), but the resulting comb included cell walls displaced from the initial guide. The outcome from for our Experiment 3 explains how this could arise. Cell walls begun on the guide ridges form cells that would be undersized, but the workers would then expand the undersized cells by continued work on each cell and therefore moving some walls away from the initial guide location.

When joining two tongues of comb of differing alignment, the location and orientation of cell walls built by bees were constrained by the sub-optimal relative positioning of cell features at the edge of each tongue. The result was that irregular cells were created along the line of contact as two tongues of comb merged (although ‘collided’ may be the *mot juste*). Cell-level irregularities in wall length and cell area at and around intersections have been found to vary smoothly across a distance of four or five cell widths either side of a junction^16^. The results of our Experiment 3 show that, when tongues first come into contact, the cells close to the junction were irregular. However, after the bees had undertaken further construction, geometric irregularities were reduced significantly. Our prediction is that size and angles are both balanced between adjacent cells. In this manner, the haphazard contact between misaligned cells initially created highly irregular cells, but continued construction and the optimisation of each cell led to reduced cell-to-cell disparity. Ultimately, this created the conditions of a gradual progression of geometries across a tongue junction^16^.

Through the experiments described in the present study, we have examined the response by bees to conditions that occur during the later stages of cell construction. The results show how the interaction between two or more cells in contact with each other equilibrates so that the cell sizes become more even and the corner angles settle so that the available angular range is evenly split between all cells meeting at a junction. Emergent from these features, the cells become hexagonal prisms with rhombic bases. In conclusion, the local geometry of honeycomb results from a self-organised process of adjustment by the bees, leading to an equilibrium of spatial resources. In future experiments, it should be explored whether the larger-scale geometry of the comb involves a form of cognition and planning^4,27^, or whether it can be similarly explained by self-organisation.

## Methods

### Hives

Experiments were conducted from May to July 2020 and June to July 2021 in England (Reigate, 51.23°N, 0.19°W). Three colonies of honeybees (*Apis mellifera*) were used, each containing a locally reared queen. Regulatory approval for this experiment was not sought as none is required for work on invertebrates. However, the colonies used formed part of the author’s apiary and were treated with the same care and welfare considerations as the other hives. Colonies were housed in Modified British National hives with open mesh floors and a single brood box containing 10 conventional frames, plus one to carry the experimental stimuli. The frames were set transverse to the entrance in ‘warm’ alignment, and the test frame was positioned at (or close to) the edge of the brood area: as the 7th frame from the front. The hives were provisioned with *ad libitum* 1:1 sucrose solution (1.0 kg cane sugar in 1.0 l water).

### Preparation of stimuli

Stimuli were fabricated using wax that had been recovered from hives within the same Reigate apiary. Flat sheets of wax, used to form the stimuli, were formed by dipping one face of a flat wooden form (75 × 40 mm) into molten wax, coating it. The thickness of the wax sheets could be varied by altering the number of immersions: three immersions yielded sheets of 0.5 mm-0.6 mm thickness, while six produced sheets of 1.0 mm-1.2 mm. Each sheet was cut into three pieces (25 × 40 mm), henceforth referred to as *tabs*, which were bonded vertically to the top bar of an otherwise empty test frame using molten wax. Three such tabs were bonded to each frame, separated by ~20 mm.

### General experimental procedure and image analysis

Experiments 1–3 were performed according to the same general protocol, as follows. At ~9 am, an experimental frame was placed into each honeybee hive, and after ~4 h it was removed to be inspected and photographed. If insufficient comb construction had occurred, the frame would be reinserted for a similar time. Frames were photographed in natural daylight using a Samsung Galaxy Camera 2 EK-GC200 (Samsung C&T Corporation, Seoul, South Korea), both initially and at each inspection point. A jig was used to hold both the frame and camera at a set distance of 390 mm. The image, which covered slightly more than the whole frame, had a resolution of 4608 by 2592 pixels. The frame, which had a total width of 333 mm, covered ~3000 pixels, resulting in an image resolution of ~9 pixels per mm. Image scale calibration was facilitated by the inclusion of a graduated measure within the field of view.

Although the mechanical jig provided linear alignment within a few millimetres and angular alignment within a few degrees, this was insufficient for comparison of wax features at the sub-cell scale. More precise alignment was achieved using software to adjust one image in each pair by making four discrete points, that were evident in both images coincident with each other. This allowed the position and shape of comb features to be more accurately compared. Photographic alignment was performed using custom software written by V.G., FormImageCompare. This software was written in C++ and developed using Microsoft Visual Studio Community 2019: Version 16.7.2, Visual C++ 2019. Image manipulation routines were supported by the library OpenCV:Version 3.3. FormImageCompare is also capable of accepting user input to mark the location of relevant features, then using those points to compute their size or orientation.

### Experiment 1

Our first prediction concerned the internal angles between the side-walls of cells: primarily, that this is not necessarily 120°. Rather, the available angular range around a junction is subdivided equally between the cells. If this space is constrained to just 180°, we predict that two cells meeting at a junction will each have an internal angle of 90°. To test this, we added a physical barrier onto our stimuli: a wax strip (2.0–3.0 mm in height by ~1.2 mm thick,) welded in place with molten wax (Fig. 2A). We predicted that when new cells are built against this barrier, the walls would meet at 90° rather than 120° (Fig. 2B, top 3 panels).

Cells would inevitably be built on the other side of the barrier as construction progressed. This barrier would ultimately be eroded, until the work taking place on both sides of the barrier would influence each other. At that stage the available angular range around the junction would be 360°. If this were to be divided equally between three co-adjacent cells, each cell would be adjusted to have an internal angle of 120° (Fig. 2B, bottom two panels).

We analysed cell construction against a straight wall stimulus in three situations: where adjacent cells (each of a depth <5 mm) had been built against one side of the barrier formed by the wax stimulus; where a wall between adjacent cells was built against a barrier formed by the wooden frame; and when construction against the wax barrier had progressed to the point where the wall was eroded and the cells were built to depths of several millimetres. This condition was typically associated with comb having covered the whole wax tab.

Our custom software, FormImageCompare, measured a sample cell’s side walls from photographs using the coordinates of points marked by the operator. In this case, a point was placed at each end of the sample cell wall, with additional marks placed to indicate the location of each arm of the wax barrier (Fig. 2C). FormImageCompare used these locations to calculate the orientation of each element and, thus, the angles between the wall and the barrier element.

### Experiment 2

Here, we tested our second prediction: that the angle between the base of a cell and its side-walls is not necessarily 120°, but that this space is distributed equally between adjacent cells where the base of the cells forms the common wall. Thus, if cells lie directly opposite to each other, rather than offset as in natural honeycomb, the mutual base will form a single face at 90° to the side-wall for each cell. Stimuli to test this prediction comprised a flat wax sheet, ~1.2 mm thick, with shallow indentations pressed into each side using a 4 mm-diameter domed rod. Such indentations prompt bees to begin cell construction at those locations^13^. The indentations were ~0.25 mm deep and 3-4 mm in diameter, and arranged as an approximation of the layout and spacing of worker cells. However, the depressions were aligned such that each was directly opposite another on the other face to encourage the construction of cells in direct opposition to each other (Fig. 4A, B). A thick wax backplane (~1.2 mm) was used to delay interactions between construction taking place on either side until the cell locations had been established, reducing the tendency of the bees to build cells in their typical alignment.

The profile of a cell base was measured using a linear array of seven depth probes (Fig. 4C). A profile gauge was constructed comprising seven depth probes bonded into a linear array so that the whole width was sufficiently narrow to fit within a worker cell without distorting the cell walls. Each element of the gauge was made using an outer barrel with a sliding inner wire. The barrel was a 23-gauge syringe needle, with outer diameter of 0.64 mm and inner diameter of 0.34 mm, from which the plastic mounting collar was removed. The inner wire was a piece of 10-gauge guitar string, diameter 0.245 mm. The seven needles were bonded into the array with epoxy resin (Araldite) forming a contour probe with an overall width of 4.9 mm. The probe wires protrude from the core of the guide tubes and therefore the span of the probes was 4.6 mm (4.9 – (0.64 – 0.34)).

The method by which base profile measurements were taken was as follows. The seven-probe wires were pushed so the ends of the wires were approximately flush with the top end of the probe barrels. The probe assembly was then gently pushed into the cell, following the line of the cell wall to keep the device orthogonal to the cell base. The sections of wire protruding at the top were then gripped with the syringe barrels to prevent further movement. The probe was withdrawn and placed alongside a 10 mm gauge. We photographed the 10 mm gauge and the lower section of the probe assembly to record the positions of the seven wires. Subsequent processing of each photograph was performed by manually marking the pixel at the bottom point of each wire as well as the top and bottom of the 10 mm gauge. Our software used the top and bottom gauge markers to compute the scale of the image (pixels per mm) and thus calculated the relative height of each probe.

The profile measurements were made of the bases of cells where the bees had built comb upon the stimuli to a depth of at least 8 mm and where there was at least 80% overlap between opposing cells and those were compared with measurements taken of bases within natural, control, cells found elsewhere within the comb (beyond the stimuli) where cells on each face overlapped by the more typical amount of approximately half a cell width.

### Experiment 3

This experiment was designed to test our third and final prediction: that initial uneven distributions of available space, both in terms of cell corner angles and cell area, will be redistributed more evenly between cells as construction progresses. When discrete tongues of comb are built independent of each other, they tend to be separated by a non-integer number of cell widths and have distinct orientations. Thus, as construction progresses and the two misaligned tongues merge together, the cells at the junction between the tongues will initially exhibit a high degree of irregularity. If our prediction is correct, then redistribution of cell sizes and corner angles should occur during further construction, reducing irregularities in cell structure by more than would be expected by chance.

**Fig. 7:**
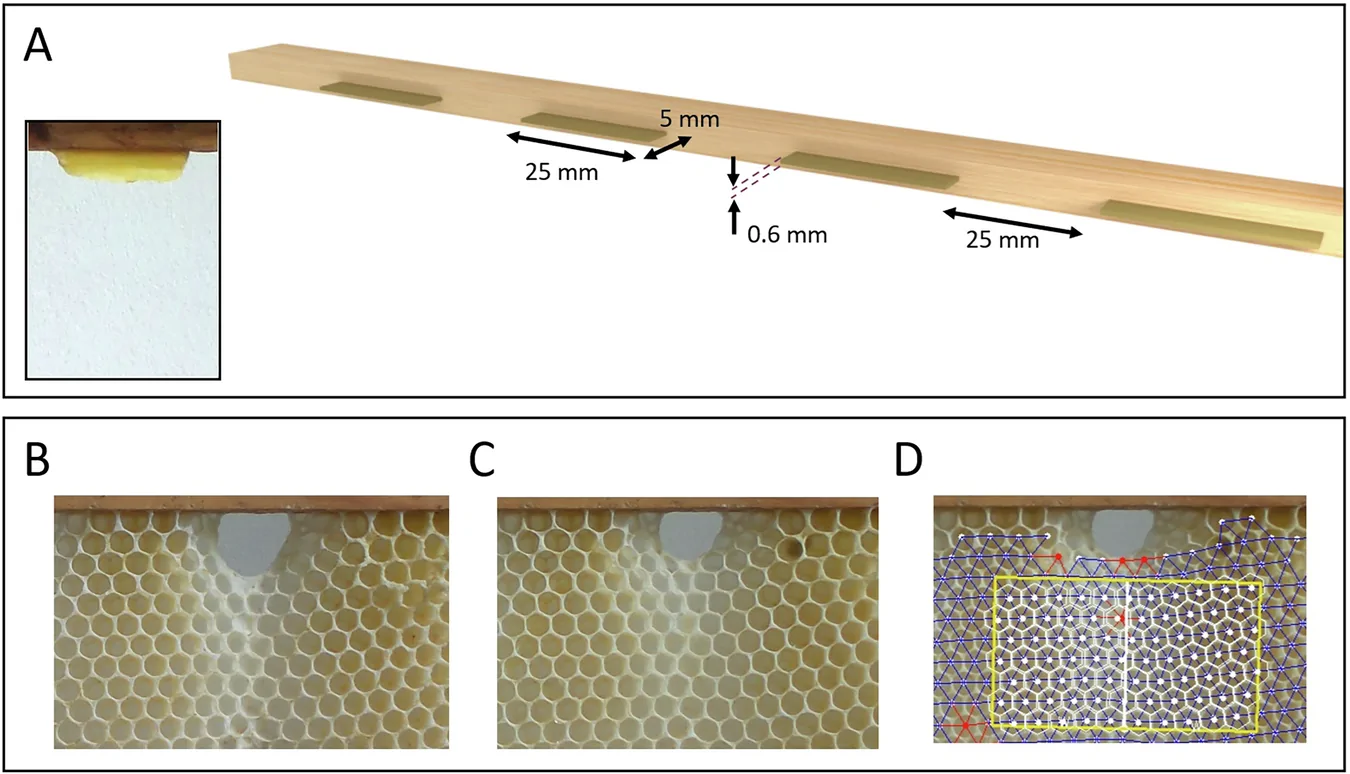
Design for Experiment 3. **A** Seed strip designed to encourage the construction of independent tongues of comb. **B** Merging of the resultant tongues of comb. Two recently-merged tongues of comb, where contact had occurred less than four hours before, and (**C**) The same comb after a further 10 hours of construction. **D** FormImageCompare software identified cell layout (approximate cell centres and adjacent cells, marked in blue) and we manually added or edited cell centres (marked in red). The yellow rectangle surrounds the cells selected for analysis, projecting 30 mm either side of the operator specified, manually input, line of contact. The selected cells, drawn in white, are defined by the corner locations, computed by software examination of the image to locate cell wall junctions and some manual edits.

The construction of several distinct tongues of comb was stimulated by preparing frames that each carried four 0.6 mm-thick strips of wax, ~25 mm by 5 mm in size. These were bonded, using molten beeswax, to the top bar of a hive frame (Fig. 7A).

For this experiment, measurements related to cell geometry were extracted using FormImageCompare software from photographs recording comb construction at two distinct stages. The image used for the initial state was where the tongues were in contact (Fig. 7B), but that had been separate in the immediately preceding photograph (no more than 4 h previously). Second-stage photographs, recording the post-merger condition of the cells, were of the comb after a further 8-12 h construction activity (Fig. 7C).

Image processing involved the alteration of brightness and contrast, followed by conversion to black and white using a localised threshold value. The threshold was computed using a Gaussian filter over an area of 63 × 63 pixels, which for these images was approximately the size of a single cell. Boundaries of black areas of the resulting black and white image (darker areas of the original image, typical of cells within the walls) were computed, then filtered, to include only those with an area typical of cells, and proximity to each other. For each of these black areas, an external hull (a shape comprising only convex external vertices) was used to derive the location of its centroid, which was assumed to represent the centre of a cell (Fig. 7D). This image processing and drawing of the resulting cell mesh was performed by our custom software in real-time. As a result, the operator could manipulate on-screen controls to adjust brightness, contrast, threshold and other values, allowing interactive optimisation of the outcome. Processing each image yielded coordinates for >95% of the cells around the tongue junctions and the remainder were then explicitly identified by the operator.

Using the centre of each cell, the software then computed the distance to each adjacent cell and thus estimated the location of a mutual wall as the mean of the centre-to-centre midpoint and the point of maximum brightness found along that line. The extent of the inter-cell wall was estimated by determining the point of maximum change of brightness along the centre-to-centre, thus providing an estimate of a cell wall’s internal face. The software assumed, as a first approximation, that cell walls lay perpendicular to the centre-to-centre line and hence computed an estimation of the location of each corner. Using the estimated location, the software used the contrast-enhanced image to search for a white band indicating a cell wall, following each into a corner to obtain an improved estimate of the location of each cell corner. A final step involved displaying these locations allowing each corner location to be inspected, and where necessary, to be modified. The resulting corner locations were used to re-compute the cell centroid and the cell attributes to be analysed (number of walls, wall lengths, internal angles and cell area).

Using the earlier of a pair of images, showing the initial contact between the tongues, the operator marked each end of each tongue merger junction. The software used these two locations to define the centre line of a zone of interest and, using these, computed a rectangle measuring 30 mm on either side of the centre line. Cells with centres contained within that rectangle were those sampled. The software also used the computed location of cells to match those in one image with those in the second, allowing analysis of cell-by-cell alterations in geometry.

### Statistical analysis

Data obtained from the images, using FormImageCompare, were subsequently processed using custom scripts written using R statistical software v4.3.2^32^. For Experiment 1, measurements were made of the angle between a cell wall and a constraining barrier. Of the two that are available, one to either side of the wall, the larger angle was selected. Additionally, a bisection was computed using the orientation of the two sides of the barrier and from this we computed a displacement angle between the bisection and the wall. A Kruskal-Wallis test was performed, using the R function kruskal.test(), to determine whether there were statistically significant differences in the value of the displacement angle between the three groups, those being cell pairs, frame cells or triple junction cells.

For Experiment 2, measurements taken of the bases using a multi-point depth gauge yielded the depth at seven points, d0–d6. The maximum displacement from the regression line through all seven points on the profile (d0–d6, Fig. 4C) was used as the measurement of flatness. The base to wall angle (one to each side of the cell) was calculated by taking the gradient of a regression line through each half of the base profile, from each of d0 and d6 to the deepest location: typically d3 or d4 (Fig. 4C). A two one-side test (TOST) comparison between the measured base inclination and the predicted value of 90° was performed. We used the smallest effect size of interest (SESOI) of 0.5 standardised mean difference, Cohen’s *d*, assuming only a moderate effect.

For Experiment 3, cells exhibiting the greatest irregularity were expected to be those close to the line of contact between tongues, therefore cells less than two cell’s width from the junction (10 mm) were used for comparison between stages 1 and 2. Cells recorded at stage 1 were matched to those recorded at stage 2 by comparison of each cell’s location. Cells were deemed to match where the location differed by less than 2.5 mm (approximately half the width of a cell). For those cells where the location could be matched between stage 1 and stage 2, the data were analysed to assess changes made to individual cells. The measures of comb regularity used a cell’s internal corner angles and its area. For cell internal angles, the metric of regularity was calculated as the variance of those values for each cell. The internal corner angle measurements were sampled for only those corners that lay *outward* of the cell centre, away from the junction, to avoid artificial duplication and ensure the single use of each datum. Our prediction was that continued construction would optimise cell layout and to test this we calculated the linear regression comparing the change in irregularity between stage 1 and stage 2 with the initial degree of irregularity.

## Data Availability

The data that support the findings of this study are openly available at: https://github.com/VinceAGallo/HoneycombResearch/tree/main/CellOptimisationPaper.
